# Supplementary material for: A study on formalizing the knowledge of data curation activities across different fields
Source: PLoS One. 2024 Apr 25;19(4):e0301772. doi: 10.1371/journal.pone.0301772 (PMC11045097; doi:10.1371/journal.pone.0301772)
Supplement: S1 File — (DOCX) [file pone.0301772.s001.docx]

(template) Questions related to data curation activities

1) Ingest

In this section, we will ask you about your data ingestion process details. This includes the identification of datasets available for registration, the procedure for receiving a deposit agreement, available media, and procedures for obtaining metadata and documentation.

2) Appraisal and Selection

In this section, we will ask you about your data appraisal and selection process details. This includes the identification of legal risks, such as the handling of personal information, arising from the characteristics of the data to be accepted, and the operation of the collection policies established by each repository.

3) Data Processing

In this section, we will ask you about your data processing process details. This includes the storage and work space environment for processing data files before publishing, the scope of work logging and who created it, matters related to the software used to handle the data, and other data processing policies.

4) Data Storing

In this section, we will ask you about the system for storing data that has been processed. This includes the workflow up to preservation and the history management in each phase.

5) Metadata generation

In this section, we will ask you about the details of handling metadata submitted by data providers. This includes the validation, modifications, and additions that the data curator will make to the submitted metadata, distribution of the metadata envisioned, and the handling of metadata tied to a specific study.

6) Access level

In this section, we will ask you about the status of your repository’s data access levels. This includes the options of access restrictions (e.g., affiliation, IP range, specific circumstances) and the types of conditions of use that are granted with description examples.

7) Long-term preservation

In this section, we will ask you about efforts for long-term preservation. This includes statements on preferred file formats, migration, format standardization/restructuring, emulation support, etc.

8) Re-evaluation and disposal

In this section, we will ask you about the metrics used to evaluate published data and how they are operationalized. This includes metrics information used as evaluation criteria (e.g., number of accesses, downloads, citations, papers linked to published data, etc.) and how the collected information is used fall under this category.

9) Other

In this section, we will ask you about the operation for conducting data curation activities. This includes the actual number of people involved in data curation activities and the allocation of specialized personnel, in addition to the number of cases processed per year and the time each process takes.
