# Supplementary material for: A study on formalizing the knowledge of data curation activities across different fields
Source: PLoS One. 2024 Apr 25;19(4):e0301772. doi: 10.1371/journal.pone.0301772 (PMC11045097; doi:10.1371/journal.pone.0301772)
Supplement: Attachment — Submitted filename: Response to Reviewers.docx [file pone.0301772.s003.docx]

Comments from the Editorial Board Member and Reviewers':

Journal Requirements:

1. Please ensure that your manuscript meets PLOS ONE's style requirements, including those for file naming. The PLOS ONE style templates can be found at

https://journals.plos.org/plosone/s/file?id=wjVg/PLOSOne_formatting_sample_main_body.pdf and

https://journals.plos.org/plosone/s/file?id=ba62/PLOSOne_formatting_sample_title_authors_affiliations.pdf

(Response)

We prepared a revised manuscript with history and a revised manuscript reflecting the changes based on the submission template.

2. Did you know that depositing data in a repository is associated with up to a 25% citation advantage (https://doi.org/10.1371/journal.pone.0230416)? If you’ve not already done so, consider depositing your raw data in a repository to ensure your work is read, appreciated and cited by the largest possible audience. You’ll also earn an Accessible Data icon on your published paper if you deposit your data in any participating repository (https://plos.org/open-science/open-data/#accessible-data).

(Response)

All data used in this study will be registered in our institution's repository (https://repository.nii.ac.jp) upon acceptance.

3. You indicated that ethical approval was not necessary for your study. We understand that the framework for ethical oversight requirements for studies of this type may differ depending on the setting and we would appreciate some further clarification regarding your research. Could you please provide further details on why your study is exempt from the need for approval and confirmation from your institutional review board or research ethics committee (e.g., in the form of a letter or email correspondence) that ethics review was not necessary for this study? Please include a copy of the correspondence as an "Other" file.

(Response)

In response to your request, we inquired with NII’s Institutional Review Board whether an ethical review was required or not. We received a response that no ethics review was required for this study. We submit the email used for the inquiry and the response as an annex.

4. Please provide additional details regarding participant consent. In the ethics statement in the Methods and online submission information, please ensure that you have specified (1) whether consent was informed and (2) what type you obtained (for instance, written or verbal, and if verbal, how it was documented and witnessed). If your study included minors, state whether you obtained consent from parents or guardians. If the need for consent was waived by the ethics committee, please include this information.

If you are reporting a retrospective study of medical records or archived samples, please ensure that you have discussed whether all data were fully anonymized before you accessed them and/or whether the IRB or ethics committee waived the requirement for informed consent. If patients provided informed written consent to have data from their medical records used in research, please include this information.

(Response)

We asked the survey institutions to cooperate in writing for the field survey. Each institution responded in writing and in the body of an email, and we surveyed only those agreed institutions. We also inserted this text in the "Materials and Methods" section.

5. Please note that PLOS ONE has specific guidelines on code sharing for submissions in which author-generated code underpins the findings in the manuscript. In these cases, all author-generated code must be made available without restrictions upon publication of the work. Please review our guidelines at https://journals.plos.org/plosone/s/materials-and-software-sharing#loc-sharing-code and ensure that your code is shared in a way that follows best practice and facilitates reproducibility and reuse.

(Response)

No code has been produced for this study.

6. We note that your Data Availability Statement is currently as follows: "All relevant data are within the manuscript and its Supporting Information files."

(Response)

We added the following descriptions to the “Data Availability Statement” section:

“All relevant data are within the manuscript and its Supporting Information files.”

=====

Additional Editor Comments:

The authors are invited to design an evaluation on the suitability of the proposed ontology to annotate data curation-related metadata. This is an important comment from Reviewer 1. Please also address all the other comments made by the two reviewers. We will be ready to assess a revised version of the manuscript.

(Response)

We attempted to annotate data curation manuals published by non-surveyed organizations based on our ontology to assess the suitability of this ontology. We added the trial results to the 'Results and Discussion' section. We also addressed all other review comments (you can find all in this file). We thank you and our reviewers for your valuable feedback.

=====

Reviewers’ comments:

Reviewer #1: The paper reviews data curation practices across a variety of disciplines, and based on this proposes an ontology to annotate data curation-related metadata. The proposed ontology is aimed to be used in an interdisciplinary setting, to allow cross-domain-compatible curation annotation.

$1:

The paper is written and structured reasonably well, although some sentences are hard to parse and I am not 100% sure I understood all details correctly. Some words are used incorrectly (e.g. "compliment" where "complement" is sensible). It is highly recommended to seek assistance of a native speaker (or a LLM) to improve the writing in future revisions.

(Response)

Thank you for your suggestion. Our manuscript has undergone English proofreading by a native speaker before submission, but they may have missed something. If you point this out to us individually, we will correct it.

$2:

Regarding the objective of the paper, I am not quite sure what the authors intended. I fundamentally endorse the stated goal of improving cross-discipline data curation annotation practices. Towards this, the authors are working on a very important and relevant problem, and their efforts are very welcome. It is my reading that the proposed ontology is aimed directly at facilitating this. However, if this is the case, I would have expected some form of evaluation on its suitability. Several examples on how it could be used would go a long way towards illustrating its relevance.

(Response)

First, this study aims to build a knowledge framework for an interdisciplinary understanding of data curation activities in different fields. Furthermore, it is hoped that the outcome will be improved annotation of data curation. To clarify this, we revised the final paragraph of the ‘Introduction’ as follows:

“This study aims to build a knowledge framework for an interdisciplinary understanding of data curation activities in different fields. For this purpose, we investigate the practices of data curation conducted in each field to interpret the tasks and procedures in different fields… This research will help to improve and facilitate cross-disciplinary data curation annotation practices.”

Next, we attempted to annotate data curation manuals published by non-surveyed organizations based on this ontology to assess the suitability of this ontology. We added the trial results to the 'Results and Discussion' section. In line with this addition, some of the descriptions in the Results and Discussion section have been modified to make them consistent. Thank you for pointing this out.

Other issues:

$3:

- The authors translated (mapped) the processes at the interviewed institutions themselves, but these translations are not available with the submission (that I was able to find). Interviewees are limited to Japan. It is unclear how much of a limitation this poses, since e.g. WEKO3 is a common factor among many institutions.

(Response)

We linked those that are documented to the rationale in Appendix 2. Those that remain internal manuals are described as 'survey participant' and are checked with the interviewee.

As you mentioned, the interviewees are limited to Japan. However, many of the data repositories interviewed operate according to international standards. Therefore, we believe that the understanding of the process can be assessed as generic.

$4:

- The technical contribution centers around translating the Data Curation Network vocabularies into an ontology, where the authors have added a structuring categorization based on control structure and process grouping. Are alternative groupings possible? It appears that the ontology is sensible and useful, but this is not discussed in detail at all.

(Response)

This study used the IPO model, the basis for information processing, to understand the logical structure. Based on this analysis, we converted the vocabulary into an ontology. Of course, applying more complex models other than the IPO model to carry out groupings is possible, and it may show different and interesting results. However, it is essential to present a framework for interpreting the vocabulary itself from an information-processing perspective, and we believe that this paper makes an essential contribution in this respect.

$5:

- I was not able to understand how consideration of staffing at the interviewed institutions contribute to the stated objectives. While this can be informative, the authors imply that it is possible to derive staffing requirements, which I find an invalid conclusion without considering further factors such as institution/repository size, submission rates, workflow overhead, etc.

(Response)

There was no intention that it would be possible to derive staffing requirements; we only want to indicate that the patterns of executing entities involved in data curation activities are diverse. We corrected the wording where it was misleading as follows. Thank you for pointing this out.

“The roles defined by each repository are different, and there is no noticeable trend in the number of appearances. Each repository’s data curation activities are conducted in different ways. For example, there are three staffing patterns in the “Data Cleaning” activities: the data holders themselves, the data curator(s), and the 2 or 3 parties working together. Some of these activities are covered by support systems or tools. For an interdisciplinary understanding of process execution, human actions and tool processes need to be viewed as different contributions to the process execution in the same actor. ~~To eliminate ambiguity in the interpretation of the activities, it is necessary to treat the process execution entity separately, either by human actions or by tool processes.”~~

$6:

- The authors critique that the Data Curation Network does not have strict hierarchy, but fail to stringently argue why this is needed in the first place. It appears that the problem the authors view is that it is not possible to create a complete process graph using on DCN information, but this is not stated in a comprehensible manner.

(Response)

As you mentioned in your review comments related to this study’s purpose, this study aims to build a knowledge framework for understanding data curation activities in different fields in an interdisciplinary way. In building the framework, definitions should be clear, comparable, and well-structured by the same components. If the same components cannot be compared, it is impossible to fully understand who performs the process, how, and what the outputs are.

We revised the text related to the objective in your other comment, and we believe this modification clarified this argument. Therefore, we did not modify this comment, but we are ready to revise it again if it still needs to be improved. Thank you for pointing this out.

=====

Reviewer #2: The study provides a valuable intellectual contribution to understanding data curation activities across domains. The paper is clearly written and presented data collection and analyses well. My only suggestions if possible would be expanding results and discussion. Future research and implications for data mangers and educators in this area would be helpful. Figures may need to be broken up into manageable parts. It is hard in a review to see how these might be presented at publication time, but there is a lot because these are complicated workflows. Overall, very important and challenging work.

(Response)

In response to your suggestions, we added suggestions for data managers, educators in the ‘Conclusion’ section. We also added future research directions in the ‘Conclusion’ section. Thank you for your valuable comments.

6. PLOS authors have the option to publish the peer review history of their article (what does this mean?). If published, this will include your full peer review and any attached files.

(Response)

Yes
